# Investigation of obesity and its related factors among Chinese medical staff: a cross-sectional pilot study

**DOI:** 10.1007/s40519-024-01643-x

**Published:** 2024-02-19

**Authors:** Guie Gao, Yuping Liu, Zhiyong Dong, Jinai He, Cunchuan Wang, Xiaomei Chen, Wenhui Chen

**Affiliations:** 1 Department of Operating Room, The First Affiliated Hospital of Jinan University; School of Nursing, Jinan University, No. 601, Huangpu Avenue West, Guangzhou, China; 2 Department of Nursing, The First Affiliated Hospital of Jinan University, Guangzhou, China; 3 Department of Metabolic and Bariatric Surgery, The First Affiliated Hospital of Jinan University, No. 613 Huangpu Avenue West, Guangzhou, China; 4 Department of Surgery Clinic, The First Affiliated Hospital of Jinan University, Guangzhou, China

**Keywords:** Obesity, Medical staff, Prevalence, Factors

## Abstract

**Background:**

Many studies have covered the prevalence of obesity in different populations. However, studies on the prevalence and predictors of obesity among medical staff are lacking. The aim of our study is to investigate the prevalence of obesity among medical staff and to identify the related predictors.

**Methods:**

Using a snowballing recruitment strategy in the form of an electronic questionnaire, a cross-sectional survey was conducted among 1201 medical staff from cooperative hospitals between January and March 2022. We designed a questionnaire to investigate the participants’ demographic, lifestyle, diet, physical activity, and work status.

**Results:**

The overall prevalence of obesity was 8.5%, with males (13.7%) having a greater incidence than females (5.7%) (p < 0.001). Multiple logistic regression analyses showed that alcohol drinking (OR, 2.34; 95% CI 1.23–4.42, p = 0.01), sugar-sweetened beverages consumed > 3/week (OR, 2.50; 95% CI 1.02–6.15, p = 0.046), and working a night shift > 1/week (OR, 2.17; 95% CI 1.02–4.61, p = 0.043) were independent predictive factors for obesity in men. For women, having midnight snack having midnight snack (OR, 2.93;95% CI 1.24–6.96, p = 0.015), good sleep quality (OR, 4.47; 95% CI 1.10–21.70, p = 0.038), and working a night shift > 1/week (OR, 3.62; 95% CI 1.73–7.57, p = 0.001) were independently associated with obesity.

**Conclusions:**

Obesity presented a low prevalence among medical staff. Alcohol drinking, drinking sugar-sweetened beverages > 3/week, and night shift > 1/week predicted a higher risk of obesity in males. In females, having midnight snack, good sleep quality, and night shift > 1/week were independently associated with obesity.

*Level of evidence*: V, descriptive study.

**Supplementary Information:**

The online version contains supplementary material available at 10.1007/s40519-024-01643-x.

## Introduction

Obesity is a primary risk factor for type 2 diabetes mellitus (T2DM), cardiovascular and kidney diseases, and several types of cancer [1, 2], making it a major public health issue worldwide. In the past decades, the prevalence of obesity has rapidly increased globally. In China, social, economic, environmental, dietary, and physical activities have experienced dramatic change. The percentage of the population that is overweight or obese has been increasing. Recent epidemiological studies have shown that 34.3% of adults over 18 years of age are overweight (BMI over 24 kg/m^2^) and 16.4% are obese (BMI over 28 kg/m^2^) [3]. The reason for this change may be that diets tend to include more animal-based foods, refined grains, and highly-processed foods. People today also have more sedentary lifestyles and reduced levels of physical activity [3]. Furthermore, stress is often related to the increased prevalence of obesity, because stress can affect eating habits [4, 5].

Medical staff, including doctors, nurses, anesthesiologists, laboratory physicians, and paramedics are among the groups of professionals who possess rich medical knowledge. They have sufficient knowledge about obesity and know how to change their lifestyle to avoid becoming overweight or obese. We hypothesized there would be a lower prevalence of obesity among medical staff. However, working in shifts is an occupational feature of the medical field [6]. Shift work makes medical staff sleep deprived, increases their workload, and leaves them with little time for physical activity. In addition, medical staff tend to be sedentary and highly stressed. Because of these factors, they are more likely to be overweight or obese. Several studies have examined the prevalence of obesity and its related factors in various fields [7–9], but there is a scarcity of data on medical staff.

Understanding the prevalence of obesity and its predictors among medical staff is useful for strengthening health management, preventing obesity and its associated comorbidities, and directing prevention efforts. However, there is yet to be any studies aimed at specially investigating the prevalence and related factors of obesity in medical staff. Therefore, the present study was designed to explore the prevalence and predictors of obesity among medical staff.

## Methods

### Study design

This cross-sectional study was conducted on medical staff from January to March 2022. Data was collected using a self-administered online questionnaire, distributed through Questionnaire Star and WeChat apps. A snowballing recruitment strategy was used: participants were asked to share the study with their friends and colleagues through their personal networks. Informed consent was requested before participants answered any questions; participants who disagreed were unable to continue filling out the questionnaire.

### Questionnaire

We designed a self-administered online questionnaire based on similar questionnaires [10–12]. The questionnaire (Online Appendix) consisted of two sections. The first section covered basic demographic data, such as gender, age, height, weight, years of work, education background, hospital level, practice category, department, and comorbidities. Another section assessed the lifestyle of participants, including diet, physical activity, and work status.

BMI was calculated by dividing participants’ height (m) in square meters by their body weight in kilograms (kg). BMI was classified as underweight (< 18.5 kg/m^2^), normal weight (18.5–24.9 kg/m^2^), overweight (range, 25.0–27.9 kg/m^2^), or obese (≥ 28.0 kg/m^2^) [13]. Smokers were defined as those who currently smoked and also those who had smoked more than 100 cigarettes in their lifetime [14]. Alcohol drinkers were defined as participants who reported consuming alcohol more than once a week. Night snacking was measured by the question: ‘Do you often eat a snack at night (more than three times a week)?’ Sleep quality was determined by asking: ‘How do you rate your sleep quality,’ for which the three responses were: good, fair and bad.

### Statistical analysis

The data was incorporated into a Microsoft Excel spreadsheet. Statistical analyses were performed in SPSS version 13.0 (SPSS Inc. Chicago, IL, USA). Data was calculated by descriptive statistics and frequency counts. Continuous data are presented as the mean ± standard deviation (SD) and categorical data are presented as a number (percentage). The groups were compared with a Chi-square test or a Fisher’s exact test for categorical data. A multivariable binary logistic regression analysis (Enter) was performed to explore possible predictive factors for obesity. A p value of < 0.05 was considered statistically significant.

## Results

### Characteristics of the study participants

A total of 1,201 medical staff completed the questionnaires, including 785 (65.4%) women and 416 (34.6%) men. Overall, the mean BMI (kg/m^2^) was 23.1 ± 3.8, the mean age (years) was 35.3 ± 8.6, and the mean amount of work experience (years) was 12.6 ± 9.1. Demographic characteristics of the participants are shown in Table 1.

**Table 1:** Characteristics of the participants

	n (%)	Means ± SD
Gender		–
Male	416 (34.6)	
Female	785 (65.4)	
Age (years)		35.3 ± 8.6
20–29	357 (29.7)	
30–39	466 (38.8)	
40–49	291 (24.2)	
≥ 50	87 (7.3)	
BMI (kg/m^2^)		23.1 ± 3.8
< 18.5	80 (6.7)	
< 24	689 (57.4))	
24–27.9	330 (27.5)	
≥ 28	102 (8.5)	
Years of work (years)		12.6 ± 9.1
≤ 5	329 (27.4)	
6–10	251 (20.9)	
11–15	228 (18.9)	
≥ 15	393 (32.8)	
Education		–
Associate degree	190 (15.8)	
Bachelor’s degree	750 (62.5)	
Master’s degree	196 (16.3)	
Doctoral degree	65 (5.4)	
Hospital level		–
Tertiary hospital	893 (74.4)	
Secondary hospital	266 (22.1)	
Primary hospital	42 (3.5)	
Personnel category		–
Nurses	458 (38.1)	
Doctors	665 (55.4)	
Others	78 (6.5)	
Department		–
Internal medicine	151 (12.6)	
Surgery	487 (40.6)	
O&G	53 (4.4)	
Pediatrics	13 (1.1)	
Emergency	42 (3.5)	
Operation room	308 (25.4)	
Others	147 (12.3)	
Comorbidities		
Hypertension	70 (5.8)	
Diabetes	27 (2.2)	
Heart disease	10 (0.8)	
Hyperlipidemia	89 (7.4)	
Hyperuricemia	101 (8.4)	

*BMI* body mass index, *O&G* obstetrics and gynecology

The analysis showed that there are significant differences in the categories of gender (p < 0.01), hospital level (p < 0.01), departments (p = 0.003), and practice categories (p < 0.01) between BMI ≥ 28 kg/m^2^ and < 28 kg/m^2^ (Table 2).

**Table 2:** Participant characteristics according to BMI categories

	≥ 28 kg/m^2^ (n = 102)	< 28 kg/m^2^ (n = 1099)	P value
Gender			** < 0.01**
Male	57	359	
Female	45	740	
Age (years)			0.094
20–29	26	331	
30–39	33	433	
40–49	34	257	
≥ 50	9	78	
Years of work (years)			0.115
< 5	26	303	
6–10	14	237	
11–15	19	209	
≥ 15	43	350	
Education			0.352
Associate degree	12	178	
Bachelor’s degree	72	678	
Master’s degree	13	183	
Doctoral degree	5	60	
Hospital level			** < 0.01**
Tertiary hospital	57	836	
Secondary hospital	39	227	
Primary hospital	6	36	
Departments			**0.003**
Internal medicine	7	144	
Surgery	60	427	
Pediatrics	1	12	
O&G	5	48	
Emergency	3	39	
Operation room	13	295	
Others	13	134	
Practice category			** < 0.01**
Nursing	32	633	
Doctor	62	396	
Others	8	70	

Bold values represent significant statistical significance

*BMI* body mass index, *O&G* obstetrics and gynecology

### Prevalence of obesity

**Fig. 1:**
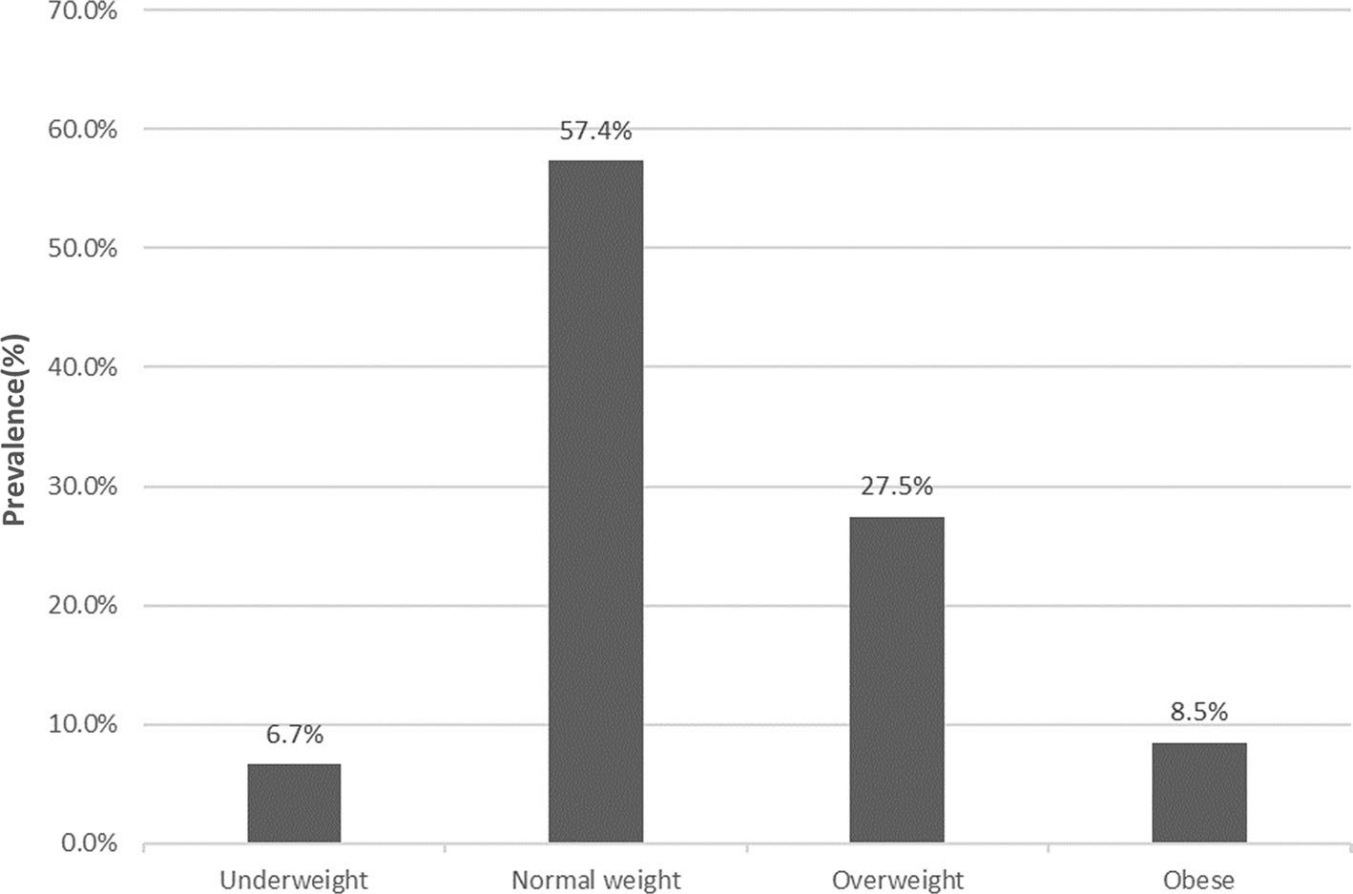
Shows prevalence of underweight, normal weight, overweight and obesity among medical staff

As shown in Fig. 1, of the 1201 participants, 8.5% (102/1201) of the medical staff fulfilled the necessary criteria to be considered obese. In male participants, the prevalence was 13.7% (57/416), whereas female patients had a prevalence of 5.7% (45/785). There was a significant difference between the prevalence of obesity in both genders (X^2^ = 22.22, p < 0.001). Over one-third (36.0%) were overweight or obese. In addition, 6.7% of participants were underweight.

**Fig. 2:**
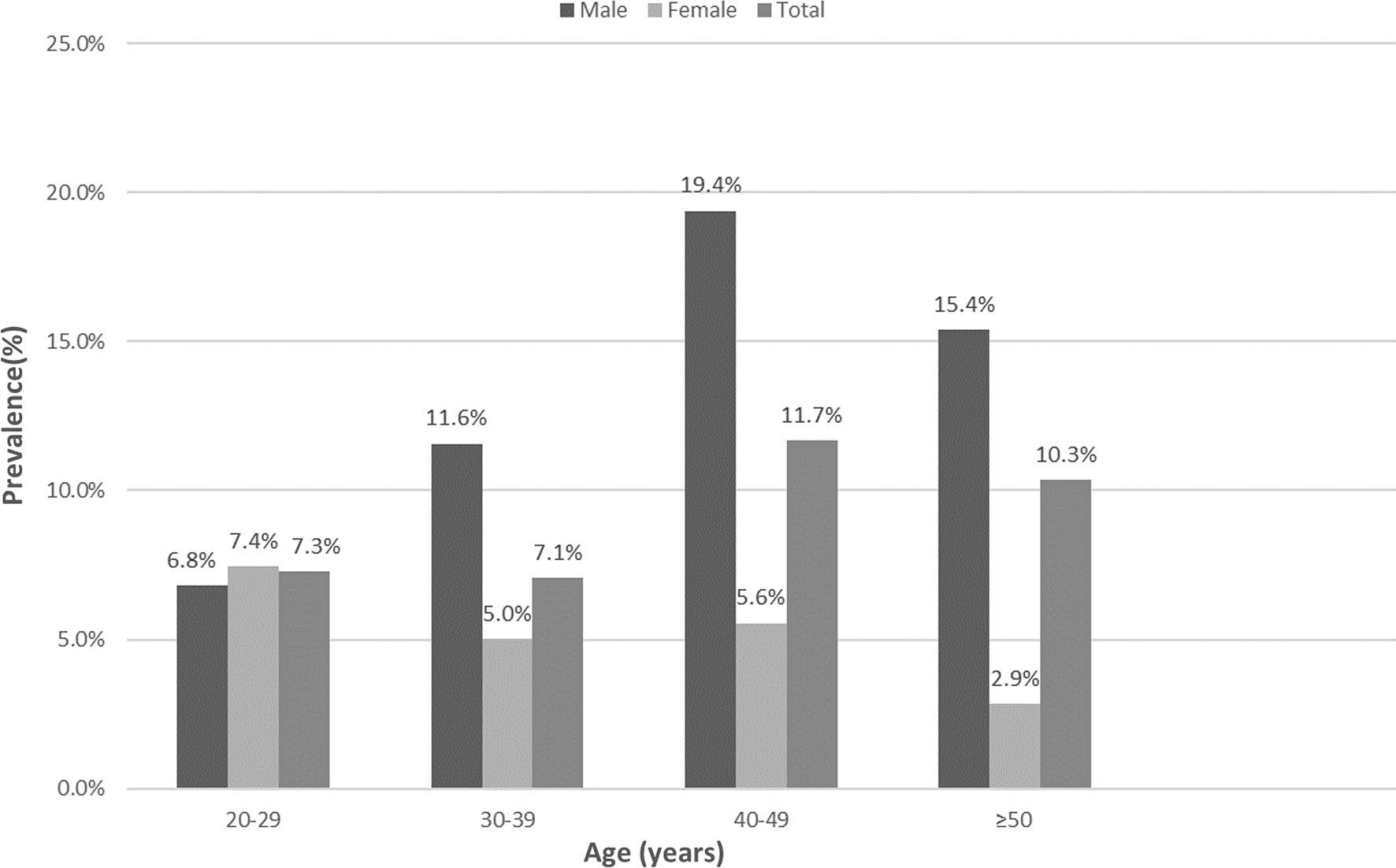
Shows the prevalence of obesity among medical staff stratified by age groups and gender

**Fig. 3:**
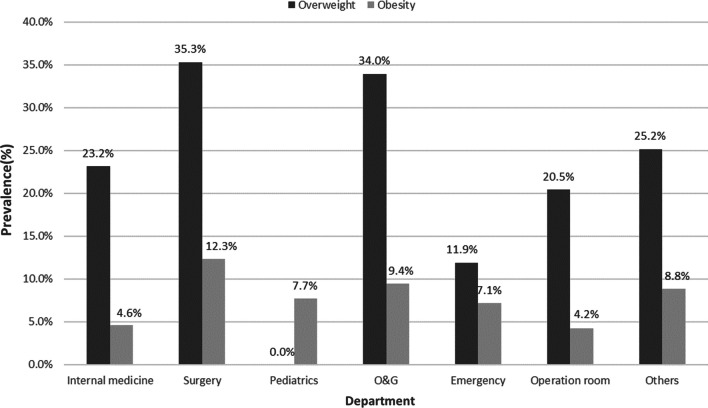
Illustrates the prevalence of overweight and obesity among medical staff stratified by departments

Men aged 40–49 years (19.4%) have the highest prevalence of obesity, while the highest prevalence of obesity in women is found between the ages of 20–29. Figure 2 shows the prevalence of obesity among medical staff organized by age group and gender. By department, medical staff in the surgical department were more likely to be overweight and obese than other departments. Figure 3 illustrates the prevalence of obesity among medical staff by department.

### Predictors of obesity

To explore the predictors of obesity, a multiple regression analysis (Enter) was performed to analyze the risk factors associated with obesity. For men, independent predictive factors for obesity were found to be drinking alcohol (OR, 2.34; 95% CI 1.23–4.42, p = 0.01), drinking sugar-sweetened beverages > 3/week (OR 2.50; 95% CI 1.02–6.15, p = 0.046), and working the night shift > 1/week (OR, 2.17; 95% CI 1.02–4.61, p = 0.043). For women, having midnight snack (OR, 2.93; 95% CI 1.24–6.96, p = 0.015), good sleep quality (OR, 4.47; 95% CI 1.10–21.70, p = 0.038), and working the night shift > 1/week (OR, 3.62; 95% CI 1.73–7.57, p = 0.001) were significantly associated with obesity (Table 3).

**Table 3:** Multivariable analysis factors associated with obesity among medical staff in different gender

	Male		Female	
	Adjusted OR (95% CI)	*P* value	Adjusted OR (95% CI)	*P* value
Age (years)				
20–29	Ref.	–	Ref.	–
30–39	0.48 (0.07–3.46)	0.463	2.29 (0.16–32.02)	0.539
40–49	0.60 (0.15–2.35)	0.460	1.96 (0.19–20.23)	0.572
≥ 50	1.15 (0.48–2.83)	0.761	2.22 (0.26–18.95)	0.466
Years of work (years)				
< 5	Ref.	–	Ref.	
6–10	1.00 (0.18–5.53)	0.99	0.96 (0.18–5.22)	0.961
11–15	0.80 (0.21–3.03)	0.743	0.57 (0.14–2.35)	0.438
≥ 15	1.12 (0.36–3.50)	0.842	0.76 (0.21–2.86)	0.702
Education				
Associate degree	Ref.			
Bachelor’s degree	1.04 (0.15–6.88)	0.981	1.57 (0.15–16.36)	0.705
Master’s degree	2.32 (0.70–7.74)	0.170	1.77 (0.19–16.92)	0.621
Doctoral degree	1.11 (0.28–4.45)	0.881	1.56 (0.14–17.53)	0.720
Smoking				
No	Ref.	–	Ref.	
Yes	0.96 (0.48–1.92)	0.897	2.43 (0.20–29.27)	0.484
Alcohol drinking				
No	Ref.	–	Ref.	
Yes	**2.34 (1.23–4.42)**	**0.01**	2.32 (0.75–7.17)	0.142
Having midnight snack				
No	Ref.	–	Ref.	
Yes	0.63 (0.22–1.78)	0.383	**2.93 (1.24–6.96)**	**0.015**
Conscious weight control				
No	Ref.	–	Ref.	
Yes	1.27 (0.63–2.57)	0.499	1.18 (0.58–2.38)	0.645
Sleep quality				
Poor	Ref.	–	Ref.	
Fair	2.15 (0.61–7.56)	0.233	3.46 (0.75–15.86)	0.110
Good	2.21 (0.62–7.88)	0.220	**4.87 (1.10–21.70)**	**0.038**
Drinking coffee				
≤ 3/week	Ref.	–	Ref.	
> 3/week	1.98 (0.53–7.40)	0.308	1.08 (0.37–3.14)	0.895
Drinking tea				
≤ 3/week	Ref.	–	Ref.	
> 3/week	1.19 (0.62–2.25)	0.604	0.90 (0.37–2.13)	0.805
Drinking Sugar-sweetened beverages				
≤ 3/week	Ref.	–	Ref.	
> 3/week	**2.50 (1.02–6.15)**	**0.046**	1.63 (0.68–3.84)	0.268
Exercise				
≤ 2 h/week	Ref.	–	Ref.	
> 2 h/week	0.87 (0.46–1.65)	0.677	1.90 (0.85–4.22)	0.118
Night shift work				
≤ 1/week	Ref.	–	Ref.	
> 1/week	**2.17 (1.02–4.61)**	**0.043**	**3.62 (1.73–7.57)**	**0.001**
Sleep time				
≤ 7 h/day	Ref.	–	Ref.	
> 7 h/day	0.83 (0.43–1.60)	0.573	0.99 (0.52–1.89)	0.975

Bold values represent significant statistical significance

*OR* odds ratio; *CI* confidence interval

## Discussion

Obesity is common in many populations, not only nurses [15, 16], but little is known about the prevalence and contributing factors of obesity in medical staff. The current study is the first of its kind to investigate the prevalence and possible predictive factors of obesity in medical staff. Our findings revealed a low prevalence of obesity (8.5%) among medical staff, and the prevalence varied by gender, age group, and department. Furthermore, we explored potential risk factors for obesity in medical staff. The results showed that drinking alcohol, drinking sugar-sweetened beverages > 3/week, and working a night shift > 1/week are independent predictors of obesity in men. In women, having midnight snack, good sleep quality, and working a night shift > 1/week were independently associated with obesity.

In this study, the overall prevalence of overweight and obese medical staff was found to be 27.5% and 8.5%, respectively, which is lower than the prevalence in the general Chinese population (34.3% and 16.4%) [3]. In addition, males had a higher prevalence of obesity (13.7%) compared to females (5.7%). A possible reason for this lower rate is that medical staff have a better understanding of obesity than the general population, even though their work means that they do not have enough leisure time to exercise and must work in shifts. Our data resembles a previous study conducted among German nurses, which reported that the prevalence of overweight nurses was 17.5%, while 5.4% were obese [16]. In a previous study on 4,878 Chinese nurses, it was found that 15.8% and 2.4% of nurses were overweight and obese, respectively [11]. For nurses in America, there is a higher prevalence: about 30% are overweight, 18.7% are obese and 5.2% are considered morbidly obese [16].

The present study found that drinking alcohol was associated with obesity among male medical staff. This finding is in line with related studies that have reported there is a positive correlation between alcohol drinking and obesity in men [17–19]. The underlying reason for this correlation may be that alcohol increases the appetite through opioid, serotonergic, and GABAergic pathways in the brain, which may lead to increased food consumption [20]. Alcohol may also lead to increased body weight [21], because alcohol inhibits fat oxidation and energy storage [22]. Consuming sugar-sweetened beverages was also found to be related to obesity in males. There is increasing evidence that frequent sugar-sweetened beverage consumption leads to a high risk for obesity [23, 24], because consumption of liquid calories decreases satiety and leads to an incomplete compensatory reduction in calorie intake at subsequent meals [25].

Notably, night shift work was identified as an independent predictor of obesity in both men and women. Similar results were reported in other studies [26, 27]. A systematic review and meta-analysis including 74,651 nurses from 11 studies demonstrated that working night shifts may play a significant role in the development of obesity among nurses [28]. Working Night shifts reduces the amount of leisure time available for physical activity and influences the quality and quantity of sleep despite supplemental sleep during the day [27, 29]. Additionally, night shift work may also change night eating habits [30]. During night shifts, employees usually eat meals or snacks high in fat and sugar and drink sugar-sweetened beverages to cope with fatigue [28]. This shows that there is an intrinsic link between night shift work and midnight snack. Therefore, it becomes clear why having midnight snack t is also closely linked to obesity.

In our study, good sleep quality was only found to be associated with obesity in females. However, previous meta-analysis revealed that poor sleep quality seems to be associated with being overweight/obese, but it also noted that a causal link between sleep quality and obesity could not be established [31]. This is because participants in the current study answered how satisfied they were with their sleep, which is a subjective experience, rather than using a more credible method of assessment such as the Pittsburgh Sleep Quality Index (PSQI). Further studies with larger sample sizes based on objective assessment are needed to better assess the relationship between sleep quality and obesity.

## Conclusions

This study showed that obesity has a low prevalence among medical staff. Drinking alcohol, drinking sugar-sweetened beverages > 3/week, and working a night shift > 1/week indicate higher risk of obesity in males; in females, having midnight snack, good sleep quality, and working a night shift > 1/week are independently associated with obesity.

## Strengths and limitations

One of the strengths of this study is that it is the first to explore the prevalence of obesity and its predictive factors among medical staff. Our study adds to the existing literature and provides new data regarding the prevalence of obesity in medical staff. However, several limitations should be acknowledged in the current study. First, the number of participants from different departments varies greatly; for example, there were only 13 participants from pediatrics, which may have led to bias in our results. Secondly, data was collected through a voluntary, self-reported questionnaire using a snowballing recruitment strategy rather than based on a population-based sample, leading to a potential selection bias. Finally, due to the cross-sectional nature of the data, the causal inferences that were made about the results need further verification. Given these limitations, further cohort studies with larger sample sizes would be valuable to better evaluate the prevalence of obesity and its predictive factors in medical staff.

## What is already known on this subject?

Several studies have examined the prevalence of obesity and its related factors in various population. To our knowledge, no known studies have examined obesity and its related factors among medical staff.

## What this study adds?

The current study adds to the data on obesity and its related factors among medical staff. Results from the current study suggest that obesity has a low prevalence among medical staff and dietary habits of medical staff may be related to obesity.

### Supplementary Information

Below is the link to the electronic supplementary material.

Supplementary file1 (DOCX 20 KB)

## Data Availability

The data used to support the findings of this study are available from the corresponding author upon request.
