# Supplementary material for: Investigation of obesity and its related factors among Chinese medical staff: a cross-sectional pilot study
Source: Eat Weight Disord. 2024 Feb 19;29(1):15. doi: 10.1007/s40519-024-01643-x (PMC10876749; doi:10.1007/s40519-024-01643-x)
Supplement: Supplementary file 1 — Supplementary file1 (DOCX 20 KB) [file 40519_2024_1643_MOESM1_ESM.docx]

**Appendix**

**Investigation of Obesity and its Related Factors Among Medical Staff: A Cross-Sectional Pilot Study**

**I. Basic information**

1. Gender □ 1. Male □ 2. Female

2. Age ____years

3. Your height: _____cm Your weight: ______kg

4. Education:

□ Associate s degree □ Bachelor’s degree

□ Master s degree □ Doctoral degree

5. Hospital level:

□ Primary hospital □ Secondary hospital □ Tertiary hos hospital

6. Department:

□ Internal medicine □ Surgery □ Pediatrics

□ Obstetrics and Gynecology □Emergency □Operation room □ Other

7. Personnel category

□Nurses □Doctors □Other

8. Years of work

□≤5years □ 6-10 years □11-15years □≥15years

9. Comorbidities (Choose more than 1)

□No □Hypertension □Diabetes

□Heart disease □Hyperlipidemia □Hyperuricemia

□Others________

**II Diet, physical activity and work status of participants**

1. During last 12 months, do you regularly smoke?

□Current smoked

□ Never (patients who had never smoked, or who had smoked less than 100 cigarettes in their lifetime).

1. Do you regularly drink any alcohol more than once a week?

□Yes

□No

1. Do you often have midnight snack more than three times a week?

□Yes

□No

4. Do you consciously control your weight?

□Yes

□No

5. How do you feel about your sleep quality?

□Good

□Fair

□Poor

6. How long do you sleep?

□≤ 7h/day

□> 7h/day

7. Do you often drink coffee more than three times a week?

□Yes

□No

8. Do you often drink tea more than three times a week?

□Yes

□No

9. Do you often drink Sugar-sweetened beverages more than three times a week?

□Yes

□No

10.How often do you work night shifts?

□≤ 1/week

□> 1/week

11. How long do you exercise a week

□≤ 2h/week

□> 2h/week

**-----END----**
